# Preparation and Properties Analysis of Chlorinated Butyl Rubber (CIIR)/Organic Diatomite Damping Composites

**DOI:** 10.3390/ma11112172

**Published:** 2018-11-02

**Authors:** Zeyuan Sheng, Siyuan Yang, Jincheng Wang, Yao Lu, Keya Tang, Shiqiang Song

**Affiliations:** College of Chemistry and Chemical Engineering, Shanghai University of Engineering Science, Shanghai 201620, China; shengzeyuanzoe@126.com (Z.S.); yangsiyuan222@163.com (S.Y.); luyao111@126.com (Y.L.); 18879475808@163.com (K.T.)

**Keywords:** diatomite, hindered phenol, chlorinated butyl rubber, damping, properties

## Abstract

In this work, a novel type of diatomite was prepared with a limited content of hindered phenol groups grafted on its hydrophobic surface. The obtained samples were characterized for their surface groups, particle morphology, pore structure, and thermal behaviors. Then, modified diatomite (MDT) was used in preparation of reinforced chlorinated butyl rubber (CIIR) composites by mechanical blending method. The powder of MDT can be uniformly dispersed in CIIR matrices and the compatibility was good. In addition, the MDT showed a positive effect on damping performance of CIIR composites. A blending ratio of CIIR/MDT = 100/10 presented the best damping performance and the damping temperature range (tan *δ* > 0.7) was extended from 60 to 70 °C. The variable temperature FTIR spectra showed the presence of hydrogen bonds between the hydroxyl groups and chloride atoms in the CIIR matrices, and a blue shift exhibited when these hydrogen bonds were dissociated. Hence, these CIIR composites provided good damping behaviors and supplied a novel and promising way for preparation of high damping rubber composites with broad temperature ranges.

## 1. Introduction

With development of science and technology, various large machines have been produced. Meanwhile, noise and friction are generated with operation of large machines. This seriously impacts the reliability and stability of the machine, not only polluting the environment, but also affecting people’s lives. Damping materials then arose, which can absorb vibrations and turn the mechanical energy into heat. Hence, they can be used to reduce the resonance amplitude and extend the fatigue life of machines [1,2].

Rubber, as an important type of damping material, has a viscoelastic structure to absorb vibration energy and convert it into heat [3,4]. Usually, high damping performance around the glass transition temperature (*T*_g_) is used to produce damping polymeric materials. Internal friction within the macromolecules is produced in the vicinity of *T*_g_, and thus mechanical energy is transformed to thermal energy and effective noise reduction is achieved. Chlorinated butyl rubber (CIIR) has better energy absorptivity and worse molecular mobility compared with any other polymeric composites [5]. It contains active chlorine and consists of isobutylene isoprene copolymer. CIIR presents a special damping behavior, that is, it exhibits a maximum on the high temperature side together with an additional shoulder on the low temperature side. However, the damping effect of CIIR is not good enough to meet the requirements, which limits its application in some fields [6,7,8].

To overcome above disadvantages, scholars have conducted more relative research using techniques such as small molecular blending which includes hindered phenol modification [9,10], organic clay intercalation [11], inorganic piezoelectric ceramics and carbon black blending [12,13], etc.; polymer blending modification including rubber and rubber blending [14,15], rubber and plastic blending [16,17], etc.; and polymer structure modification including copolymerization [18,19], interpenetrating polymer network [20,21], multilayer co-extrusion method [22], etc. Mechanical blending has been explored as a simple and feasible method to obtain materials with new performances [23]. When two or more kinds of incompatible materials are blended, a novel polymer-based composite with a microscopic phase separation structure can be fabricated [24]. Thus, damping materials may be prepared from a practical method by introducing additives into polymer matrices. Furthermore, it has been reported that hydrogen bonding interactions between additives and polymers may improve damping properties of composites [9,10,25,26,27].

Diatomite (DT) is a type of biological and silicon-containing sedimentary rock. It is very cheap and abundant in resources. In addition, it is porous and possesses the characteristic of lightness. The main component of DT is amorphous SiO_2_, with a small quantity of feldspar, quartz, mica, etc. Many studies focus on the adsorption properties of DT, which may be promoted by reacting with other materials adhered to its surface [28,29]. In addition, some researchers investigated the application properties of modified DT in rubber composites. Wu et al. [30] used modified diatomite to reinforce different types of rubbers by ultrasonic dispersion technology. Through detailed study of the mechanical properties, it is revealed that silica may be replaced by DT and some rubbers may be filled by these powders. Liao et al. [31] adopted surface modification of diatomite by stearic acid. Its effect on the mechanical properties for natural rubber/styrene butadiene rubber composites (NR/SBR) is studied. Results show that the surface of diatomite exhibited hydrophobic and the tensile properties are improved due to a physical adsorption between stearic acid and diatomite.

However, until now, the modification of DT based on hydrogen bonds and the research on its damping performance and mechanism in rubbers has not been conducted. In this work, modified DT was prepared using hindered phenol and was added as functional fillers to CIIR rubber matrix. The properties of modified CIIR such as cure characteristics, tensile properties, and dynamic mechanical performance were investigated in detail.

## 2. Experimental

### 2.1. Materials

Diatomite, industrial grade, was received from Shanghai Shengdiya Company (DT, Shanghai, China). KH560, CH_2_OCHCH_2_O(CH_2_)_3_Si(OCH_3_)_3_, industrial grade, was obtained from Chemical Agent Company (Shanghai, China). Antioxidant 245, C_34_H_50_O_8_, industrial grade, was supplied by Shanghai Anhui Chemical Company (Shanghai, China). Xylene, anhydrous ethanol, and chloroform were provided by Sinopharm Chemical Reagent Co., Ltd. (Shanghai, China). Chlorinated butyl rubber (CIIR, Exxon 1066) was obtained from Exxon Mobil Chemical Industry Company (Spring City, PA, USA). Rubber additives were obtained from the Market (Shanghai, China).

### 2.2. Preparation of Modified Diatomite (MDT)

A certain amount of diatomite was put into a crucible, and then cured in a muffle furnace for 4 h at a temperature of 400 °C, and the calcined DT was obtained. The calcined DT was treated over 100-mesh sieve to get pretreated DT. Eight grams of pretreated DT were added into a solution of 100 mL of ethanol and water with a ratio of 1:1. Then, acetic acid was added into above mixture, and the pH value was adjusted to 4. After acidification, 10 g of the silane coupling agent, KH560, were added slowly into the system. The KH560 modified DT, KH560-DT, was obtained after washing and drying. Finally, 10 g of antioxidant 245 were dropped into the mixture of KH560-DT, and it was stirred for 2 h with a temperature keeping at 70 °C for 12 h. After this reaction, the product was washed, filtered and dried at 60 °C for 24 h to get the final product: modified diatomite (MDT).

### 2.3. Preparation of CIIR Composites

CIIR gums were blended with different rubber additives on a double roller plasticator (Shanghai Second Rubber Machinery Factory, XK-1600, Shanghai, China). The polymer was mixed with different amounts of DT or MDT for 30 min, and thus different CIIR matrices were obtained. Curing was performed at 170 °C for 30 min, after which different elastic vulcanizates were prepared. Table 1 presents the formulation used for application of DT and MDT. Here, CIIR refers to pure CIIR, CIIR/MDT-5 refers to the CIIR added with 5 phr of MDT, and the other samples were named CIIR/MDT-10, CIIR/MDT-15, and CIIR/MDT-20, correspondingly.

### 2.4. Characterization

Polarizing microscope (PLM, XPF-300) and scanning electron microscopy (SEM, Hitachi S-2150) were used to observe surface morphology of different diatomites. The samples used in SEM observation were gold coated with an IB-3 Ionic sputtermeter. Fourier transform infrared spectroscopy (FTIR, Avatar 370) was operated for determination of the groups in different materials. The powders were pressed with KBr for testing in transmission mode. Sixty-four scans were used to obtain spectra with good signal-to-noise ratios. X-ray diffraction (XRD, Bruker) data were performed using an X-ray diffractometer. The X-ray beam used was nickel-filtrated Cukα (λ = 0.154 nm) radiation which was operated at 50 kV and 150 mA. The data were obtained from 5° to 80° (2θ) with an operating rate of 2°/min. N_2_ adsorption test was carried out with a ASAP2460 model of the whole automatic physical adsorbent equipment. Curing behavior of the rubber composites was tested by rubber vulcanizing machine, MDR-2000E. The tensile properties of different CIIR composites were tested by GT-TCS-2000 tensile testing machine. Thermogravimetric analysis (TGA, Linseis PT-1000) was obtained under nitrogen atmosphere with a flow rate of 5 × 10^−7^ m^3^/s. The temperature started from room-temperature to 800 °C with an increasing rate of 10 °C/min. The thermal conductivity was investigated by DRL tester. In this test, the size of the sample area was 706.89 mm^2^. Dynamic mechanical analysis (DMA, USA Q800) was measured in a liquid nitrogen condition with a heating rate of 3 °C/min. The tension mode was used with a fixed frequency of 1 Hz. The samples were cut into a size of 40 mm × 10 mm × 20 mm. The damping factor, storage modulus, and loss modulus of the composites were obtained with a temperature range from −60 °C to 80 °C. Temperature-dependent FTIR spectra were investigated on a Nicolet iS50 FTIR spectrometer conjunct with a heating equipment. The sample was heated at a rate of 5 °C/min in nitrogen atmosphere. The temperature for data collecting was set at 25, 50, 75, 100, 150, 200, and 250, respectively.

## 3. Results and Discussion

### 3.1. Analysis of Modified Diatomite (MDT)

**Figure 1:**
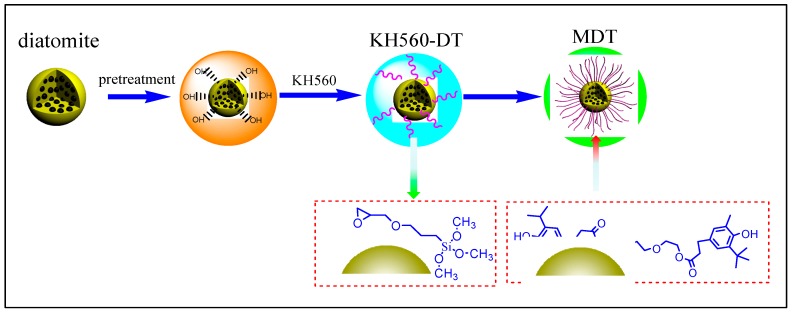
Scheme of modification process of MDT.

**Figure 2:**
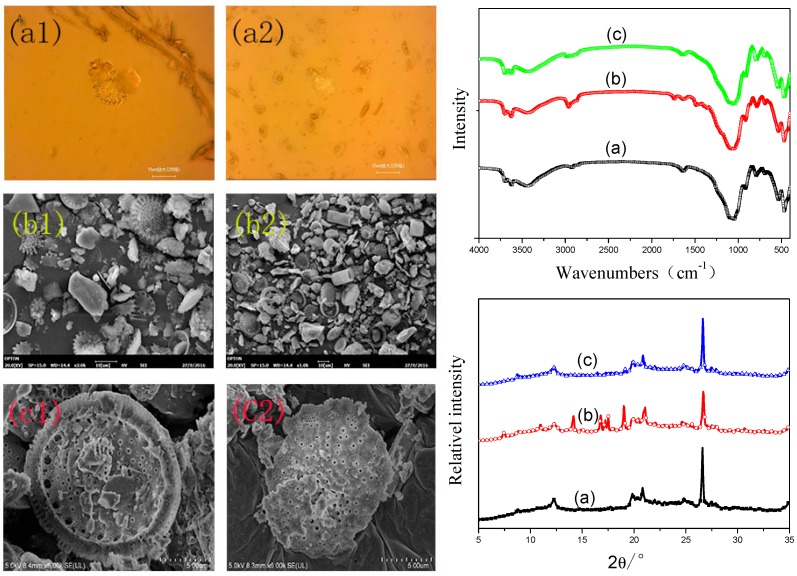
PLM, SEM, FTIR, and XRD of different diatomites: (**a**) original DT; (**b**) KH560-DT; and (**c**) MDT.

The procedure for preparation of MDT is presented in Figure 1. As illustrated, the pretreated DT was first modified by KH560 with a condensation reaction, and thus organic DT was fabricated. Then, hindered phenol was used to modify this intermediate by condensation reaction, and modified diatomite (MDT) was thus obtained [31]. The morphology of the different diatomites was observed by PLM and SEM, as shown in Figure 2. For original DT (Figure 2(a1,b1,c1)), the micro-morphology of DT was typical of round shaped sieve. However, it showed bad dispersion status in aqueous or methanol system. Additionally, the aggregates can be formed between diatomites as a nexus. For MDT (Figure 2(a2,b2,c2)), as expected, the surface modification had little effect on morphology. Its surface was slightly convex and the surface pores became fuzzy, accompanying a phenomenon of wrapping. This illustrated that the surface properties of DT were changed. Moreover, the improved dispersion degree in solvent proved that the surface polarity of MDT was increased. In addition, according to SEM, the original DT had a larger powder size, and the particles were aggregated together. However, after modification, the particle size of DT was significantly smaller, and this was attributed to removing of impurities together with coating of the modifier which may reduce the agglomeration of DT [30].

The FTIR spectra of the original and modified DT are depicted in Figure 2. In the curve for DT, strong infrared absorption peaks were shown at 1090 and 800 cm^−1^, owing to the stretching and bending vibrations of Si–O–Si bonds. Moreover, the peaks between 3500 and 3600 cm^−1^ were related to the –OH groups in DT. Compared with original DT, the intensity of –OH stretching vibration in the vicinity of 3600 cm^−1^ was decreased gradually for KH560-DT. This indicated that Si–OH groups in the hydrolysis product of KH560 had a good condensation reaction with original Si–OH groups in DT. The wavenumbers between 2800 and 3000 cm^−1^ were the stretching vibration of –CH_3_/CH_2_ groups, which indicated that the modifier was deposited on the surface of DT [32]. For MDT, these absorption peaks became sharp after modification by hindered phenol which owned many methyl groups. The absorption peaks between 1500 and 1680 cm^−1^ were –C=C and –C=O groups, respectively, illustrating that some hindered phenol groups were attached on the surface of DT in a certain degree.

The XRD patterns of original and modified DT are presented in Figure 2. First, the original DT was found to be amorphous, and the main peaks appeared at 19–22° and 26°, which were ascribed to the amorphous phase of DT [30]. Second, new peaks appeared between 12° and 17° in the curve of KH560-DT, and they were attributed to effect of silane coupling agent on the structure of DT. This phenomenon revealed that the crystallinity of DT may be changed due to covering of the coupling agent over its surface. Third, compared with that of KH560-DT, some peaks disappeared in the curve of MDT, illustrating the initial crystallization component was destroyed together with formation of some novel structure in MDT.

**Figure 3:**
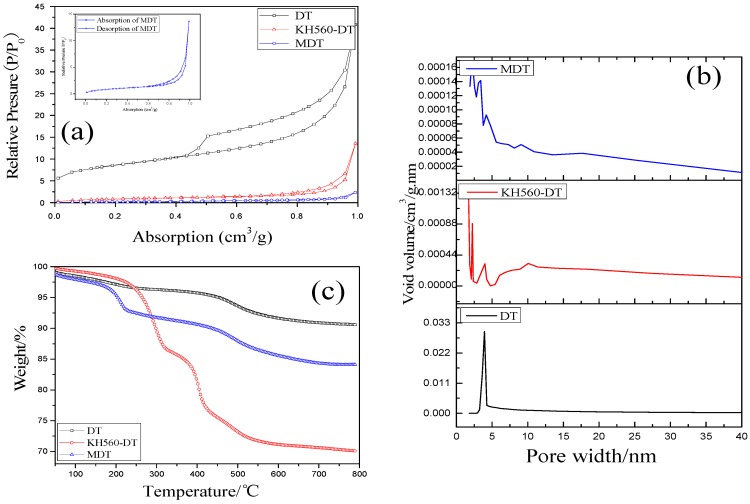
BET (**a**); porosity distribution (**b**); and thermal stability (**c**) of different types of DT.

As shown in Figure 3, all three samples exhibited a hysteresis loop, and this phenomenon was ascribed to capillary condensation. When a material has a mesoporous structure and Kelvin radius of hole, it can show capillary pressure and a corresponding phenomenon of adsorption and desorption. In addition, the pore size distribution of these diatomites was not very uniform, and they were not ordered materials [32]. The hysteresis loops of DT belonged to H3 type, and the adsorption capacity of the high-pressure side was relatively large [31]. This illustrated that some mesoporous structures existed in this powder. The adsorption capacity of MDT was significantly decreased, and was far less than that of the original DT. This revealed that some large molecular substances were grafted on the surface of DT, and many the original holes were covered. The above analysis showed that the modification process was carried out, not only on its surface but also into its pores.

Table 2 presents the specific surface area and average pore diameter of diatomites. Usually, the specific surface area of DT is 33–65 m^2^/g. The pore size of DT used in our experiments was relatively small, with an average size of 26 cm^3^/g. After modification, the surface area of MDT decreased significantly, which was ascribed to the introduction of modification agents into the holes. Moreover, the pore size of MDT was increased. During process of modification, many small holes were destroyed, while many big holes were formed. This led to a sharp decline in surface area and an increase of average pore size [33].

TGA curves of different diatomites are also shown in Figure 3. Compared with DT, the thermal stability was improved before 250 °C and decreased after 250 °C after modification by KH560. The weight loss between 200 and 800 °C was about 8%, which was probably due to degradation of the coupling agent. For MDT, its degradation occurred in three successive steps. The first step started at about 50 °C. This was related to the degradation of organic materials attached over the surface of DT. The second step occurred between 220 and 500 °C, and the third step was from 500 to 780 °C. The weight loss in the temperature range of 500–780 °C for MDT was about 5%. This can be attributed to the decomposition of carbonaceous materials existed in the residue.

### 3.2. Cure and Tensile Properties of CIIR Composites

**Figure 4:**
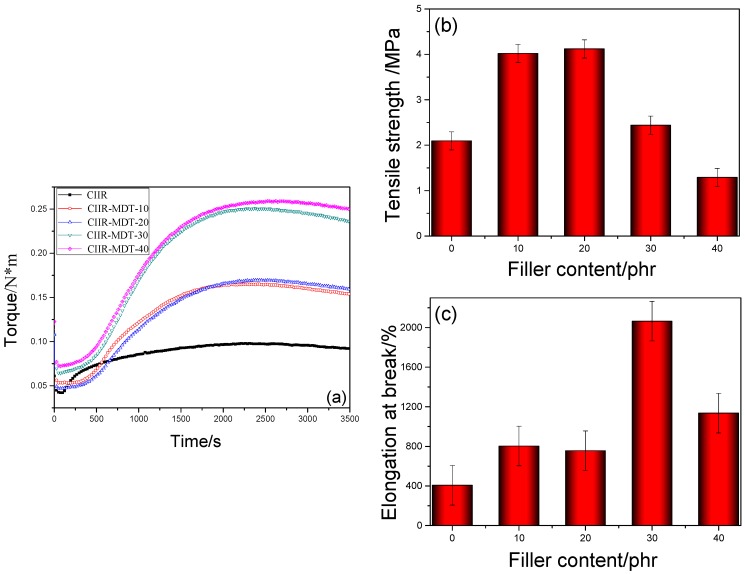
Cure curves (**a**); tensile strength (**b**); and elongation at break (**c**) of different CIIR composites.

Figure 4a shows the influence of MDT on cure characteristics of CIIR composites, and the corresponding data are summarized in Table 3. It was shown that MDT had a great difference on the cure behavior compared with that of the original DT. With addition of MDT, the torque, M_H_, was obviously increased during the vulcanization process. This may be ascribed to the high activity of MDT which possessed many available particle surfaces to interact with some components in the crosslinking system such as sulfur and accelerators. Moreover, it was noted that the rubber composites containing MDT exhibited a prolonged T_10_ and T_90_, that is, a longer scorch time and a higher energetic effect of vulcanization. Moreover, the curing rate showed an almost increasing trend. The hydroxyl groups in hindered phenol and epoxy groups in KH560 may interact with the matrix of neat CIIR, and thus the cure reaction may be affected at appropriate temperatures such as 160 °C. Therefore, this powder seemed to be active in the vulcanization process [34].

The mechanical properties of CIIR composites with different amounts of MDT are given in Figure 4. With an increasing ratio of MDT, mechanical properties of the CIIR vulcanizates such as tensile strength and elongation at break showed different trends. The tensile strength (Figure 4b) reached the highest value, 4.1 MPa, when the amount of MDT was 20 phr. It was 95% higher than that of pure CIIR (2.1 MPa). This may be ascribed to the stiff nature of DT, which was used to reinforce rubber materials if compatibility was well [35,36]. However, this property became worse due to poor compatibility of the rubber composites. In Figure 4c, the elongation at break was increased with an increase of MDT’s content. The composite with 30 phr of MDT owned the highest value (2000%), which was five times the value of pure CIIR (400%). This toughing effect of MDT may be ascribed to the physical interactions, such as covalent cross-linking, hydrogen bonds, and van der Waals forces, together with entanglement, embedding, etc. [37]. The cracker and reformation of hydrogen bonds during stretching process may give the best elongation at break. However, the elongation at break of CIIR elastomers was weakened when more amounts of MDT were added. This could be attributed to the aggregation of this additive which decreased crosslinking density of the polymer matrix.

### 3.3. Thermal Properties of CIIR Composites

**Figure 5:**
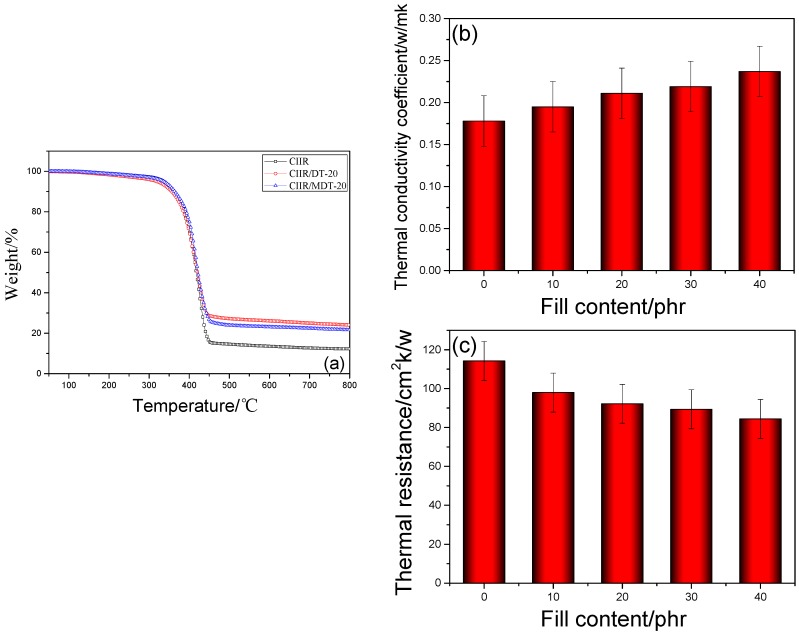
Thermal stability (**a**); thermal conductivity coefficient (**b**); and thermal resistance (**c**) of different CIIR composites.

Figure 5a presents TGA curves of pure CIIR and its composites with different types of DT. The thermal degradation of pure CIIR comprised of one main loss step. This process started at 220 °C and reached the highest rate of weight loss at about 425 °C. The decomposition mainly resulted from thermal degradation of molecular chains in CIIR. Moreover, thermal stability of CIIR may be affected by the polymer matrix, additives used, and the physical interactions among them. As a type of filler, DT and MDT showed some effects on this property, and the two types of composites presented similar thermal behavior. CIIR/DT-20 and CIIR/MDT-20 composites displayed higher decomposition temperatures than that of virgin CIIR. It is interesting to note that MDT had different effects on CIIR matrix. When the temperature was lower than 200 °C, the doping content of MDT showed little effect on the thermal decomposition performance of CIIR matrix. However, the presence of MDT increased the thermal stability of the composites between 200 and 450 °C compared with that of DT. In addition, the thermal decomposition and residual mass of CIIR/MDT composites were decreased when the temperature was higher than 450 °C. The mechanism for thermal decomposition of MDT was almost the same as that of DT due to the same shape of TG curves. Thus, it can be concluded that addition of MDT can improve thermal stability of CIIR composites [38].

The polymer possessed an intrinsically low thermal conductivity and was a good insulator for various applications. However, all damping materials demand relatively high thermal conductivity. The thermal conductivity and resistance of different CIIR composites are presented in Figure 5b,c. As can be seen, their thermal conductivity coefficient increased with the increase of MDT’s fraction, and the value of CIIR/MDT-40 was about 30% higher than that of pure CIIR matrix. This high thermal conductivity may be attributed to appropriate ratio of the small powders embedded in CIIR composites, in which a random conductive bridge or network was properly developed. The small MDT powders formed the main thermally conductive paths in the matrices, and they acted as the connection components to enhance the corresponding thermal behavior [39,40].

### 3.4. Dynamic Mechanical Properties of CIIR Composites

**Figure 6:**
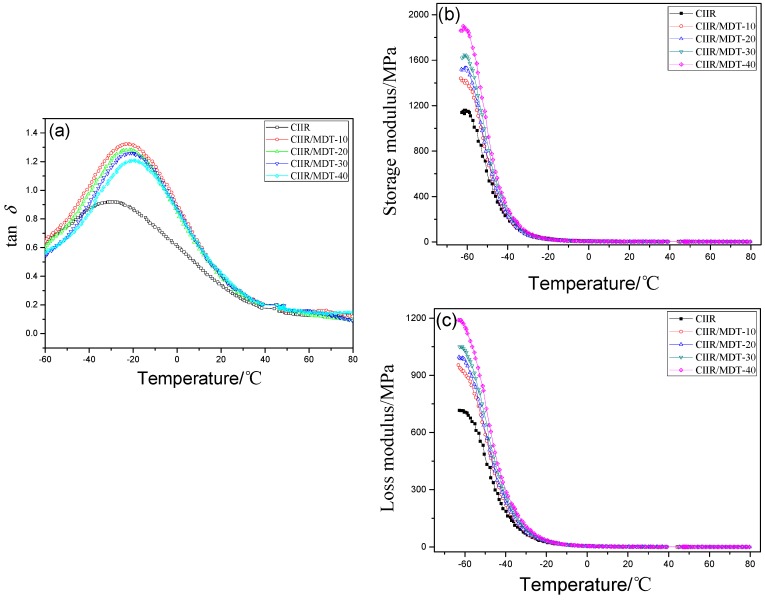
Dynamic mechanical properties of CIIR systems: (**a**) tan *δ*; (**b**) elastic modulus; and (**c**) loss modulus.

The variation of loss tangent (tan *δ*), storage modulus (*E*′), and loss modulus (*E*″) of the virgin CIIR and CIIR blends are presented in Figure 6. Tan *δ* refers to the ratio of dissipated energy to stored energy in one deformation cycle, and it reflects the internal and external frictions. Therefore, a higher tan *δ* indicates a better damping property of the composites. Tan *δ* of CIIR and its composites was 0.92, 1.32, 1.27, 1.26, and 1.21, respectively (Figure 6a). That is, after adding 10 phr of MDT, the damping factors improved about 43% compared with that of neat CIIR. This may be resulted from the special structure of MDT. The concentration and hydrogen bonds structure in hindered phenol of MDT had a great effect on the dynamic mechanical properties of CIIR composites. Thus, the damping behavior of the composite was improved. The peak generally referred to the glass transition temperature (*T*_g_) of the matrix. It is noted that the location of the peak was shifted to high temperatures due to incorporation of MDT, that is, from −31 °C for pure CIIR to −22 °C for the composites, which was totally different from most systems incorporated with other inorganic fillers. In addition, only one tan *δ* peak appeared in each composite, and this illustrated a good compatibility between MDT and CIIR relating to the results mentioned above. Achieving an effective damping performance in diversified practical applications often requires the damping materials to possess wide temperature ranges. As the amount of MDT was increased, the damping temperature ranges (tan *δ* > 0.7) were improved from about 60 (−60 to 0) to about 70 °C (−60 to 10). Under external force, the hydrogen bonds in MDT were activated, breaking and rebuilding alternately, and thus more energy was consumed and a new relaxation peak at higher temperature appeared.

The storage modulus *E*′ (Figure 6b) increased with the increase of MDT. This was due to the presence of DT with high rigidity and a higher degree of crosslinking in presence of the hindered phenol. E’ remained almost invariable above *T*_g_, and a rubbery plateau was observed with an increase of temperatures. The drop of the storage modulus revealed an enhancement in the flexibility of the composites. The E’ curves for CIIR/MDT composites showed only one transition, and gradually shifted toward higher temperatures, indicating no phase separation occurred and strong H-bonds interactions existed in the complicated networks. Figure 6c depicts the curves of loss modulus *E*″ for CIIR/MDT composites. Corresponding to the relaxation peaks of hydrogen bond in tan *δ* curves, it can be seen that E’’ of the composite tended to increase with the MDT’s content [41,42].

### 3.5. Reinforcing and Damping Mechanisms of MDT in CIIR Composites

**Figure 7:**
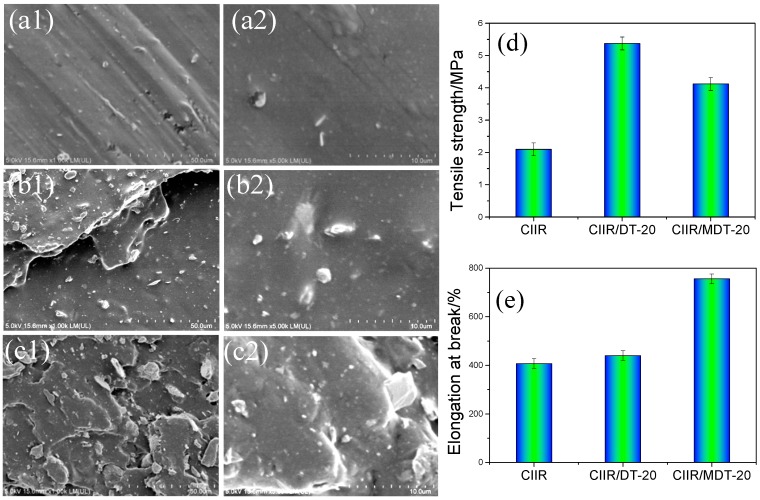
SEM of different CIIR systems: (**a1**,**2**) pure CIIR; (**b1**,**2**) CIIR/DT-20; and (**c1**,**2**) CIIR/MDT-20; (**d**) tensile strength; and (**e**) elongation at break.

Figure 7 presents SEM images of the fractural surfaces of RTV composites with different types and amounts of diatomite. Pure RTV matrix showed an almost smooth surface with some holes embedded in it (Figure 7(a1,2)). It could be found that the addition of DT had some impact on the surface morphology and micro-structure of the composite matrix (Figure 7(b1,2)). The fractural surface exhibited a featured characteristic of sea–island morphology due to immiscibility between DT and CIIR matrix. The DT phases were almost evenly dispersed in the CIIR matrix, which was attributed to a good compatibility between fillers and polymer matrix. After addition of MDT, the fractural surface of composites owned the spots with improved densities, which demonstrated a transition to ductile but more toughing structure (Figure 7(c1,2)). In addition, the MDT modified CIIR composite exhibited some pore-like structures. This was ascribed to a large particle size of the filler–rubber combination which was larger than that of the unmodified one. This large particle size also made the length of the links between the macromolecules chains in the rubber matrix become longer [43]. Meanwhile, under external force, the pores can be extruded, deformed, and mechanical energy was turned to thermal energy. As a result, high damping behavior can be achieved. Furthermore, compared with that of CIIR/DT-20, the corresponding decreased tensile strength (Figure 7d) and increased elongation at break (Figure 7e) of CIIR/MDT-20 can be supported by above relative morphology.

**Figure 8:**
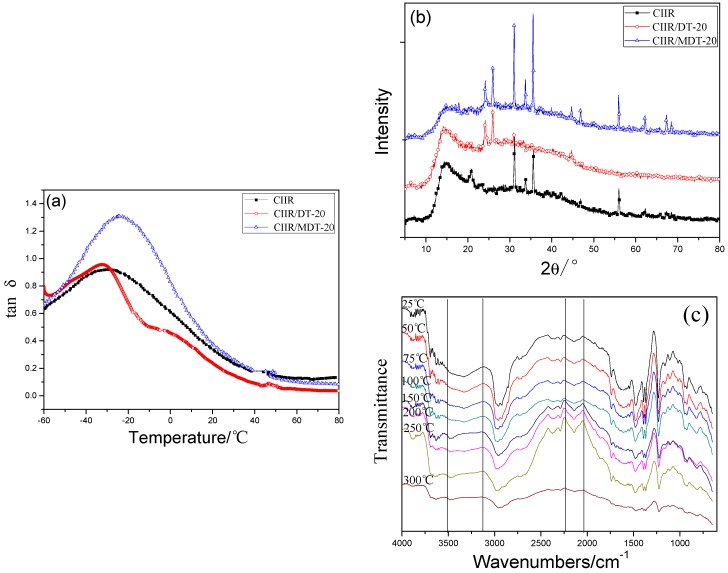
Damping mechanism of CIIR systems: (**a**) tan *δ*; (**b**) XRD; and (**c**) in situ variable temperature FTIR spectra of CIIR/MDT-20.

Figure 8a presents tan *δ* curves of CIIR elastomers with different diatomites. The original DT had a minor impact on damping behaviors of the elastomer. However, the value of tan *δ* was obviously increased after modification by hindered phenol. This was caused by the hydrogen bonding interactions between hydroxyl in hindered phenol and chloride atoms in CIIR. Meanwhile, the break and recombination of hydrogen bonding resulted from external force led to further consumption of energy and an obvious enhancement of tan *δ*. In Figure 8b, it can be found that some peaks appeared or disappeared at 20–40°, and these peaks were ascribed to the CIIR matrix and its additives. After addition of 20 phr of original DT, some peaks disappeared at 32–36° and new peaks appeared at 23–26°, which were attributed to the special structure of DT and its influence on the crystallinity of polymer matrix. However, after incorporation of MDT, both peaks of CIIR and DT appeared in the XRD curves, which illustrated the bad compatibility between DT and the polymer matrix. Figure 8c presents the temperature dependent FTIR spectra of CIIR/MDT-20 to reveal the hydrogen bonds in the composite. At 25 °C, the FTIR spectra of the hydroxyl groups exhibited a wide peak at 3300 cm^−1^, and this may be ascribed to a random network of hydrogen bonds within MDT or between CIIR polymers and hydrogen bonds in MDT. With a gradual increase of temperature, the wavenumbers gradually shifted to the region of higher frequency. This was a type of blue shift which was related to the dissociation of the hydrogen bonds [44]. Moreover, with an increase of the temperature, the absorption peaks became widened. Especially, when the temperature exceeded 150 °C, the characteristic peaks at 3500–3610 cm^−1^ for free hydroxyl groups were gradually apparent. This was resulted from destroying of the H-bonded hydroxyl groups when the temperature was higher than the melting point of the composite.

**Figure 9:**
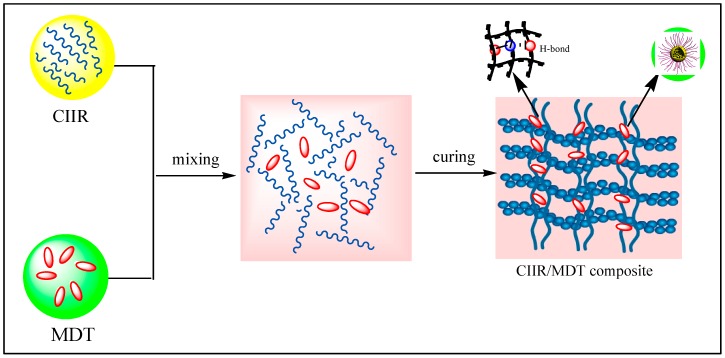
Fabrication process of CIIR damping composites based on MDT.

Figure 9 depicts fabrication process of the damping composites based on the blends. MDT was dispersed uniformly in the matrix of CIIR ascribing to mechanical shearing force which resulted from mechanical mixing. Then, compounds with organic–inorganic network structure were formed, in which MDT was used as a connection to rubber molecular chains. Here, the rubber molecular chains only adhered to the surface of MDT, and crosslinking reaction had not taken place. Finally, more bonds from weak physical force were formed and an interconnected structure thus originated. In this structure, by hydrogen bonds and Van der Waals attraction forces, many molecular chains were entangled and absorbed on the surface of MDT [32]. Therefore, MDT possessed good interfacial adhesion with CIIR matrix, which played an important role in promoting damping performance of these composites.

## 4. Conclusions

This work successfully prepared a novel type of MDT which was an effective additive for improving the dynamic mechanical performance of CIIR composites. In comparison with original DT, the novel MDT possessed different morphology, groups, structures, and properties.

The incorporation of MDT dramatically improved the tensile strength and elongation at break from 2.1 MPa and 400% for pure CIIR to 4.1 MPa and 2000% for CIIR/MDT composites, respectively. In addition, the addition of MDT improved the thermal stability and thermal conductivity of CIIR composites. In addition, the damping factors, tan *δ*, were improved about 43% compared with that of pure CIIR and the damping temperature ranges (tan *δ* > 0.7) were also widened. The mechanism for its better damping performance was attributed to the hydrogen bonds among the matrix, which may lead to further dissipation of mechanical energy and a dramatic enhancement of loss factors.

## Figures and Tables

**Table 1:** Formulation of different CIIR composites prepared by DT and MDT.

Component	phr (Parts per Hundred of Rubber)
CIIR	100
zinc oxide	3
tetramethyl thiuram disulfide	1.5
stearic acid	1.5
*N*-phenyl-β-naphthylamine	2
diben zothiazole disulfide	2
sulfur	3
DT/MDT	0, 5, 10, 15, 20

**Table 2:** Specific surface area and average pore size of different types of diatomites.

Samples	Specific Surface Area (cm^3^/g)	Average Pore Size (nm)
Original DT	26.37	9.15
KH560-DT	3.67	22.90
MDT	1.04	13.52

**Table 3:** Cure data of different CIIR composites.

Samples	T_10_/min	T_90_/min	M_L_/N·m	M_H_/N·m	Cure Rate, VC1
CIIR	2.40	26.23	0.04	0.10	4.20
CIIR/MDT-10	7.57	25.72	0.05	0.17	5.51
CIIR/MDT-20	7.82	30.00	0.05	0.17	4.48
CIIR/MDT-30	8.00	27.23	0.06	0.25	5.20
CIIR/MDT-40	7.80	28.17	0.07	0.26	4.91
